# Pelvic tumoral actinomycosis

**DOI:** 10.1590/0037-8682-0396-2025

**Published:** 2026-02-09

**Authors:** Elif Gündoğdu

**Affiliations:** 1 Eskişehir Osmangazi University, Faculty of Medicine, Department of Radiology, Eskişehir, Turkey.

**FIGURE 1:**
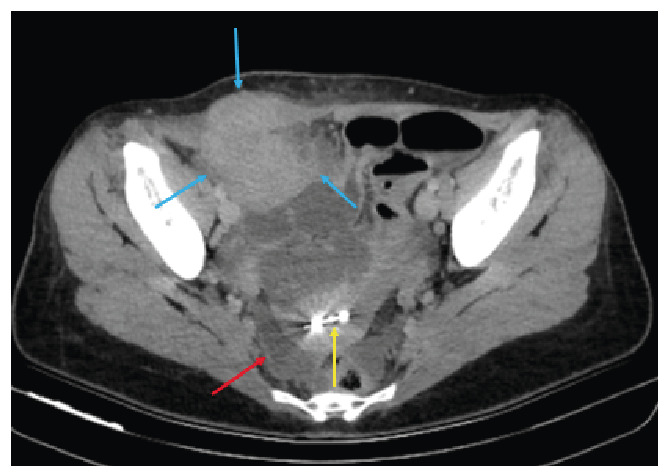
Axial pelvic CT demonstrates an abdominal wall mass (blue arrows) extending into the adjacent intra-abdominal and subcutaneous tissues, accompanied by pelvic fluid accumulation (red arrow). An intrauterine device (IUD) is seen inside the uterine cavity (yellow arrow).

**FIGURE 2:**
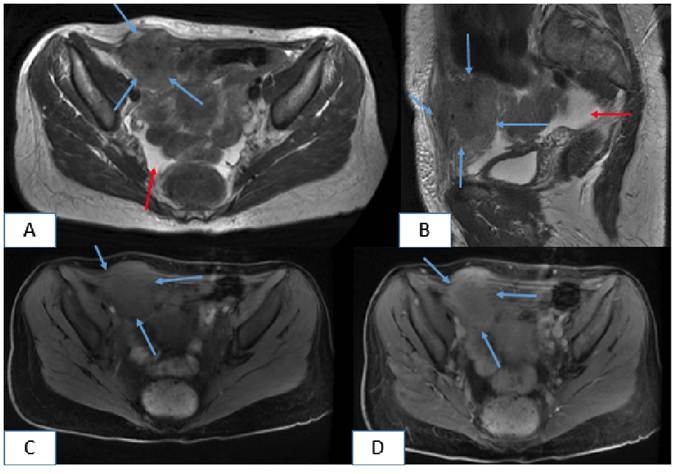
Axial **(A)** and sagittal **(B)** T2-weighted MRI reveals a hyperintense abdominal wall mass (blue arrows) extending into the adjacent intra-abdominal and subcutaneous regions, with associated pelvic fluid accumulation (red arrows). Axial precontrast **(C)** and postcontrast **(D)** T1-weighted MRI shows the enhancement of the mass.

A 45-year-old woman presented herself to the emergency department with a 15-day history of pelvic pain, fever, and fatigue. Her medical history was unremarkable, and she had no menstrual irregularities. An intrauterine device (IUD) had been implanted five years earlier. Her vital signs were within normal limits. However, laboratory test results indicated monocytosis (1.04 × 10^3^/µL), and urinalysis revealed pyuria (24 white blood cells/high-power field [HPF]) and hematuria (11 red blood cells/HPF). An initial ultrasound performed at an external center detected pelvic fluid and a pelvic mass. Owing to the presence of this mass and a preliminary diagnosis of gynecologic malignancy, the patient underwent computed tomography (CT), followed by magnetic resonance imaging (MRI). CT revealed a hyperdense mass involving the right rectus abdominis muscle and extending into the adjacent intra-abdominal and subcutaneous tissues, along with pelvic fluid accumulation. The IUD was observed inside the uterine cavity (Figure 1). MRI confirmed the normal appearance of the uterus and ovaries. It also revealed a well-enhanced mass extending from the right rectus muscle into the adjacent intra-abdominal and subcutaneous compartments, accompanied by complex pelvic fluid (Figure 2). Subsequently, the patient underwent surgical intervention. Histopathological examination revealed inflammatory granulation tissue and *Actinomyces* species. No evidence of neoplasia was found. Actinomycosis is a chronic, suppurative, and granulomatous infection caused by *Actinomyces*, a Gram-positive anaerobic bacterium^1^. Pelvic involvement is rare, accounting for approximately 3% of all human actinomycosis cases^1^. Disruption of the uterine mucous membrane by an IUD is a known predisposing factor^2^. *Actinomyces* infections that form mass-like lesions may mimic pelvic or abdominal wall malignancies. Being a diagnostic challenge that is particularly relevant to patients with IUDs, the possibility of such infections should be carefully considered in this patient population.
